# Multiple Sites of Type II Site Ligand (Luteolin and BMHPC) Regulation of Gene Expression in PC-3 Cells

**Published:** 2012-12

**Authors:** Barry M. Markaverich, Mary Vijjeswarapu

**Affiliations:** *Department of Molecular and Cellular Biology, Baylor College of Medicine, One Baylor Plaza, Houston, Texas 77030, USA*

**Keywords:** luteolin, BMHPC, type II sites, c-FOS, p21, siRNA knockdown, prostate cancer

## Abstract

Type II [^3^H]estradiol binding site ligands including luteolin (a naturally occurring bioflavonoid) and synthetic compounds such as 2,6-bis((3-methoxy-4-hydroxyphenyl)methylene)cyclohexanone (BMHPC) inhibit normal and malignant prostate cell (PC-3, LNCaP, DU-145) proliferation *in vitro* and *in vivo*. Type II sites represent a binding domain on histone H4 possibly involved in an epigenetic mechanism for controlling gene transcription. Treatment of PC-3 human prostate cancer cells with luteolin or BMHPC modulated the expression of a number of genes in the epidermal growth factor receptor signaling pathway (EGFRSP) and cell cycle pathway (CCP). Pronounced stimulation (400-2000% of control) of c-FOS and p21 RNA expression was observed, suggesting that these were primary sites of action. Both compounds also caused irreversible G2/M arrest (*p*<0.001). siRNA’s for c-FOS or p21 reduced the RNA expression of their respective targets by 85-95%, with minimal effects on cell proliferation. Furthermore, neither siRNA alone (single knockdown), or in combination (double knockdown), blocked luteolin or BMHPC inhibition of PC-3 cell proliferation. Thus, although c-FOS and p21 are known to modulate the expression of genes in the ESGRSP (EGFR, SOS, GRB2, JNK1, MKK4, RasGAP) and CCP (CCNA2, CCNE2, CDC25A, CDKN1A, CDKN1B, p27, PLK1) involved in the regulation of cell proliferation by luteolin and BMHPC, the c-FOS and p21 siRNA knockdown studies reported here suggest that c-FOS and p21 may be secondary bystanders in the overall response to these ligands in the regulation of PC-3 cell proliferation.

## INTRODUCTION

Two classes of [^3^H]estradiol binding sites (type I or type II) exist in normal and malignant cells (1-5). Type I sites (classical ERα or ERβ) are estrogen and/or anti-estrogen binding transcription factors that regulate gene transcription (6). Nuclear type II sites bind [^3^H]estradiol with a lower affinity (Kd»20 nM) than the ER and exist at basal levels (<3000 sites/cell) in non-proliferating cells. In reproductive tissues such as the uterus (3, 7) or prostate (8-11), the quantity of type II sites is increased (10-30-fold) by estrogenic hormone administration, resulting in the stimulation of DNA synthesis and cell proliferation. Alternatively, malignant tissues undergoing uncontrolled DNA synthesis and cellular proliferation contain high concentrations of type II sites, an observation consistent with their diminished regulatory control (5, 12).

Although type II sites bind estrogen in exchange assays, their affinity for [^3^H]estradiol (Kd»20 nM) is insufficient for binding serum levels (low pg/mL) of steroid under physiological conditions (13). However, methyl-p-hydroxyphenyllactate (MeHPLA) was subsequently identified as the endogenous ligand for the type II site (14). Nuclear type II sites bind MeHPLA with very high affinity (Kd 1-5 nM), and this binding interaction is likely involved in the control of an important cell growth regulatory pathway. MeHPLA is a bioflavonoid or tyrosine metabolite found in all normal tissues and serum (14-16) and appears to be a critical missing link between the lower cancer incidence in humans consuming higher quantities of fruits and vegetables (17-19). Occupancy of type II sites by MeHPLA blocks estrogen stimulation of rat uterine growth and inhibits breast cancer cell proliferation (14). Thus, it is not surprising that naturally occurring bioflavonoids (luteolin, quercetin, etc.) mimic MeHPLA as cell growth regulating agents (4, 20-23).

The biological activity of MeHPLA is regulated by MeHPLA esterase, an enzyme stimulated by estradiol in the rat uterus and constitutively expressed at a high level in malignant cells (21, 24, 25). This esterase hydrolyzes MeHPLA to HPLA (p-hydroxyphenyllactate), the corresponding free acid. HPLA does not bind type II sites with high affinity (Kd>200 nM) and does not inhibit normal or malignant cell proliferation (14). A MeHPLA-esterase-induced deficiency in MeHPLA in malignant cells results in a higher level of unoccupied type II sites and the loss of regulatory control (14-16, 21, 24). Thus, MeHPLA-esterase stable type II site ligands including luteolin, quercetin, and BMHPC, bind to nuclear type II sites with high affinity and inhibit breast cancer (14, 24, 26), pancreatic cancer (27), prostate cancer (23), colorectal cancer (28) ovarian cancer (28), lymphoblastoid (29), and leukemia (30) cells *in vitro* and *in vivo* suggesting this is a component of a universal control mechanism.

We recently identified nuclear type II sites as histone H4 (31-33). Subsequently, cRNA microarray analysis and real-time PCR (QPCR) studies with RNA from PC-3 human prostate cancer cells identified a number of luteolin-regulated genes in the EGFRSP (EGFR, c-FOS, SOS, GRB2, JNK1, MKK4, RasGAP) and in the CCP (CCNA2, CCNE2, CDC25A, CDKN1A (p21), CDKN1B (p27), PLK1) possibly involved in the anti-proliferative response to luteolin (34, 35). Chromatin immunoprecipitation studies (ChIP assays) further suggested that luteolin alters the acetylation state of histone H4 associated with the PLK1 gene promoter in PC-3 cells (35). These discoveries suggest that type II sites control cell proliferation through an epigenetic mechanism involving ligand control of histone H4 modification (acetylation/deacetylation/methylation/phosphorylation, etc.). The studies described in this manuscript evaluate the effects of luteolin and BMHPC on the RNA expression of genes in the EGFRSP and CCP in PC-3 cells regulated by these type II site ligands as they relate to cell cycle progression and cell proliferation. Gene knockdown studies with siRNA’s for c-FOS and p21 were performed. The studies provide insight into the molecular mechanisms involved in the regulation of prostate cancer cell proliferation by naturally occurring (luteolin) and synthetic (BMHPC) ligands that could be used to model new anticancer drugs. Both of these compounds reduce the weights of the prostate in normal mice, inhibit PC-3, LNCaP and DU-145 prostate cancer cells *in vitro* and inhibit the growth of prostate cancer xenografts in nude mice (22, 23). Consequently, mechanistic studies with both compounds could be used in the design of novel type II site ligands for the control of malignant cell proliferation. Since type II sites (histone H4) are ubiquitous, these findings will extrapolate to a multiplicity of cancer cell types, further accentuating the value of these studies.

## MATERIALS AND METHODS

### Reagents and Materials

Luteolin was purchased from Indofine Chemicals (Hillsborough, NJ).

BMHPC was synthesized in our lab (4). Quantitect Primers for the genes assayed by QPCR were purchased from Qiagen, Inc. The Dharmacon On Target + Smart Pool siRNA’s (c-FOS, p21, non-target) were from Thermo Scientific (Lafeyette, CO).

### PC-3 Cell Growth and Experimental Conditions

Stock cultures of PC-3 human prostate cancer cells were maintained in T-75 flasks containing 10 mL of DMEM-F12 media supplemented with 10% fetal calf serum (FCS) and 1% penicillin-streptomycin (23). For these experiments 4.4 × 10^5^ PC-3 cells were seeded into 6 well test plates (pyrogen, free, RNA/DNA free, RNase/DNase-free, TPP test plates; Midsci Laboratory Equipment and Supplies, St. Louis, MO) and grown in 3 mL of DMEM-F12 containing 10% fetal calf serum. Twenty-four hours after plating (Day 0), the exponentially growing cells were treated with various test reagents (luteolin, BMHPC, siRNA’s, etc.) and grown for additional periods of time (24-144 hours) as described in the text and figure legends. Controls (cells grown in DMEM-F12 media alone) were used to evaluate vehicle (ethanol, DMSO, Lipofectamine 2000, etc.) effects on PC-3 cells. Luteolin was added to the cells in 2-5 μL of ethanol and BMHPC was added in 2-5 μL of DMSO. These two vehicles maximized solubility of the compounds under these *in vitro* conditions. In either case, the term vehicle was used interchangeably for either ethanol or DMSO depending upon whether luteolin or BMHPC were added to the culture media.

At the termination of each experiment, cells were harvested by mild trypsinization (22) for RNA isolation, flow cytometry and/or cell number determinations. For QPCR studies, harvested cells were collected and stored in RNA-later. Attached cell number was monitored by hemocytometer counts based upon either trypan blue dye exclusion (26) or crystal violet dye uptake (36). The latter assay consists of staining the cells with 0.2% crystal violet dissolved in 20% ethanol, washing the fixed monolayer’s with water, and reading the absorbance at 560 nm in water: MeOH:EtOH (5:1:4).

### Assessment of BMHPC Effects on EGFRSP and CCP Gene Expression

Microarray studies published by our laboratory identified specific genes in the EGFRSP (EGFR, c-Fos, SOS, GRB2, JNK1, MKK4, RasGAP) and CCP (CCNA2, CCNE2, CDC25A, p21, p27, PLK-1) in PC-3 cells whose expression is modulated by luteolin (35, 37). The studies described here expanded our scope to include evaluation of BMHPC effects on these EGFRSP and CCP genes. BMHPC should modulate the same genes regulated by luteolin in both pathways since these two ligands have equivalent type II site binding affinities (Kid»5 nM) and cell inhibitory properties in PC-3, Du-145 and LNCaP cells *in vitro* and *in vivo* (4, 22, 23, 26, 38). Studies were thus performed to compare effects of BMHPC and/or luteolin on the RNA expression of these EGFRSP and CCP genes in PC-3 cells. We were particularly interested in c-FOS and p21 since the magnitude of the response of these two genes (fold-stimulation) identified them as key targets for luteolin in PC-3 cells (35). Therefore, the effect of BMHPC on their expression was evaluated in the present study for comparision.

For these studies, stock cultures of PC-3 cells grown as described above were seeded into 6 well plates (4.4 × 10^5^ cells/well). Twenty-four hours following plating (time 0), the media was changed and attached cells were treated for 24 hours with 4 μL vehicle (controls) or 10 μg/mL BMHPC (in 4 μL vehicle). At this time, RNA was prepared from the harvested PC-3 cells and subjected to QPCR (37) to assess BMHPC effects on EGFSP and CCP gene expression. QPCR analyses were performed on a minimum of three RNA pools for each treatment group. That both luteolin and BMHPC inhibited PC-3 cells *in vitro* and when grown as xenografts in nude mice (22, 23, 37), is consistent with the effects of these ligands on these EGFSP and CCP genes which are involved in PC-3 cell proliferation (35, 37, 39).

### Reversibility of Luteolin and BMHPC on c-FOS or p21 Gene Expression

To assess and compare the reversibility of luteolin or BMHPC on c-FOS or p21 gene expression, triplicate wells of PC-3 cells for each control or experimental group were treated on Day 0 with luteolin or BMHPC (5-10 μg/mL) dissolved in vehicle (controls). The cells were grown an additional 48 hours in the presence of vehicle, luteolin or BMHPC to assess the effects of continuous luteolin or BMHPC treatment on c-FOS or p21 gene expression (RNA). A separate series of wells containing PC-3 cells were treated for 24 hours with vehicle (controls), luteolin or BMHPC and then changed to fresh media and grown in the absence of luteolin or BMHPC for an additional 24 hours to study drug removal effects on c-FOS or p21 expression. The cells from either group (continuous or drug-removed) were collected as described in Figures 2 and 3 and RNA was prepared for assessment of treatment effects on c-FOS or p21 expression by real-time PCR (QPCR) as described below.

### RNA Preparation

The methods for the preparation of RNA from PC-3 cells were as described by our lab (26, 37). At the termination of each experiment, PC-3 cells from the various treatment groups were washed with PBS and collected with 0.25% trypsin-0.02%EDTA. Following 3-minute incubation, the trypsin was inactivated with 10 mL of media containing 10% FCS. For each of the triplicate RNA pools, approximately 5.0 × 10^6^ cells from the collected wells were centrifuged (2000 rpm × 5 minutes) in RNAse/DNAse free tubes, resuspended in 1mL of PBS plus 4 mL of RNAlater (Qiagen) and stored at -20°C. The frozen cells were thawed on ice, collected by centrifugation and lysed by resuspension in 0.6 mL of RTL (Qiagen) containing β-mercaptoethanol. The cells were disrupted by centrifugation through Qiashredders (18,000 × g × 2 minutes) and the pass-through was diluted with an equal volume of 70% ethanol and loaded onto RNeasy spin columns. The columns were eluted with RW1 wash buffer followed by RNAse-free DNase digestion to remove residual DNA according to the manufacturer’s instructions. Purified total RNA was eluted from the RNeasy spin columns with 50 μL of RNAse-free water following 5-minute incubation at 22°C. RNA integrity is routinely verified on an Agilent 2100 Bioanalyzer (35).

### Real-Time Quantitative Polymerase Chain Reaction (QPCR)

Pre-validated commercially available primers for the various EGFSP and CCP genes were purchased from Qiagen, Inc. QPCR was performed using the MyiQ SYBR Green Supermix (Bio-Rad Laboratories) and quantified on MyiQ Single Color Real-Time PCR Detection System using MyiQ Optical System Software, version 2.0 (Bio-Rad). Validation of each primer pair for each of the EGFRSP or CCP genes was accomplished by generating standard serial dilution and melt curves on cDNA prepared from RNA isolated from PC-3 cells. Reaction efficiencies of 90-110% and correlation coefficients of >0.995 were routinely obtained. Melt curves demonstrating a single reaction product with an appropriate melting temperature confirmed that primer dimerization was not contributing to the signal. Results from quadruplicate QPCR runs on triplicate pools of RNA from controls (ethanol, DMSO, Lipofectamine 2000), luteolin, BMHPC, or siRNA (c-FOS, p21 or Scrambled) treated cells were normalized to 18S RNA. Products of the optimized reactions were analyzed by agarose gel electrophoresis to ensure that the size of the amplicon corresponded to the data provided by Qiagen for each primer pair (35).

### Cell Cycle Analysis by Flow Cytometry

Flow cytometry studies were performed as described by our lab (22, 23). PC-3 cells (4.4 × 10^5^) were plated in 6 well plates in DMEM/F12 media and grown for 24 hours. At this time, the cells were treated with 10 μg/mL luteolin or BMHPC. After 24 hours of treatment, the cells were collected by trypsinization, washed twice in cold PBS and fixed in 70% cold ethanol and stored at -20º. Ethanol suspended cells were centrifuged and re-suspended in 25 ug/ml of propidium iodide and 50 ug/ml of DNase free RNase. Stained cells were kept for 20 min. at 22ºC and cell fluorescence determined by flow cytometry (BDFACS CANTOII; BD Biosciences, Franklin Lake, NJ) in the Cytometry and Cell Sorting Core Facility at Baylor College of Medicine. The flow cytometer was set for excitation with blue light at 488 nm and PI emission at red wavelength at 633 nm. FACS Diva software (version 6.1.3, BD Biosciences) that de-convolutes DNA content frequency histograms was used for data analyses. Each experiment was replicated three times.

### Effects of c-FOS or p21 siRNA on c-FOS and p21 Gene Expression and PC-3 Cell Proliferation

siRNA knockdown studies were performed to validate luteolin or BMHPC effects on c-FOS or p21 genes as they relate to downstream regulation of luteolin or BMHPC inhibition of PC-3 cell proliferation. Predesigned and validated Dharmacon On-TARGETplus SMARTpool siRNA’s to c-FOS (cat # L-003265-00-0005), p21 (cat # L-0033471-11-0005) and non-target sequence (cat #D-001810-10-05; scrambled siRNA) were purchased from Thermo Scientific. A series of time and dose studies were performed (not shown) for each siRNA to define conditions for maximum knockdown of c-FOS or p21 and to validate specificity of the knockdown. Maximum knockdown (90-95%) of either c-FOS or p21 was obtained following 48-hour treatment with 30 nM concentrations of c-FOS or p21 siRNA’s. Scrambled siRNA failed to substantially suppress the expression of c-FOS or p21 RNA as expected. This treatment protocol (48 hour treatment with 30 nM siRNA for c-FOS or p21) was used for all studies with the individual siRNA’s. The combination knockdown studies utilized 30 nM concentrations of c-FOS siRNA and p21 siRNA (total siRNA concentration 60 nM) as described in the text and figure legends.

For the siRNA knockdown, triplicate wells of PC-3 cells were seeded for each vehicle control or treatment group. Twenty-four hours following plating, the attached cells were treated with 30 nM siRNA for c-FOS, p21 or non-target (scrambled) sequence. siRNA’s were added to media in 8-10 μL of Lipofectamine 2000 Reagent (Life Technologies, Carlsbad, CA 92008). Forty-eight hours following vehicle (Lipofectamine 2000) or siRNA treatment, the cells were collected in RNA-later and RNA was prepared as described above. siRNA effects on the expression of c-FOS or p21 RNA were assessed by QPCR. We also assessed the effects of c-FOS or p21 siRNA knockdown on luteolin or BMHPC inhibition of PC-3 cell proliferation. PC-3 cells were treated in triplicate with c-FOS siRNA, p21 siRNA or Scr siRNA for 48 hours exactly as described above. After 48 hours of siRNA treatment (time 0), the cells were treated for an additional period of time with vehicle (controls) or 10 μg/mL luteolin or BMHPC as described in the text and figure legends. Cell number for triplicate wells for each of the various treatment groups was expressed as a percent of the vehicle controls (100%). RNA was prepared from these cells at the termination of the experiment for QPCR analysis.

Because of the complexity of these siRNA studies, which required the assessment of luteolin or BMHPC effects on cell proliferation at long times (4-6 days) following 48 hours of siRNA treatment, we focused only on c-FOS and p21 RNA expression (QPCR). siRNA effects on the c-FOS or p21 protein expression (Western blots) proved either to be too difficult to interpret, or there were too few cells remaining for sufficient protein isolation following luteolin or BMHPC treatment. Protein expression does not usually correlate with RNA expression for the majority of mammalian genes (40) and this problem is further accentuated when the response (antiproliferative effects of both luteolin and BMHPC) occurs at long times (3-4 days) following 48 hours of siRNA treatment. Changes in these proteins in dying cells at long times following drug treatment as they relate to changes in RNA occurring 6-8 days earlier are difficult to evaluate with respect to response. Additionally, c-FOS and p21 expression and histone H4 synthesis (type II binding sites available for luteolin and BMHPC binding) are cell cycle-coupled events that further complicate correlating RNA and protein expression response profiles in these experiments. For these reasons, we focused on siRNA effects on RNA expression as they relate to luteolin or BMHPC regulation of PC-3 cell proliferation with the assumption that at some point in time, the change in RNA expression following luteolin, BMHPC or siRNA treatment would be reflected by a corresponding change in protein expression. The primary goal of the studies described in this manuscript was to define luteolin and BMHPC effects on the expression of the EGFRSP and CCP genes, and define the role of c-FOS and p21 in the long-term effects of luteoin and BMHPC on PC-3 cell proliferation. This was accomplished.

### Statistical Analyses

The statistical analysis of QPCR data was as described in detail (37). Each experiment was repeated at least 3 times. Thus, the QPCR analyses were performed on quadruplicate aliquots of at least 3 pools of RNA from replicate controls or treated PC-3 prostate cancer cells and normalized to 18S RNA. The QPCR data (mean ± SEM) were analyzed by Analysis of Variance (ANOVA) and Tukey’s test on the treatment means or by a two-tailed T-test utilizing Instate (GraphPad Software). Similarly designed replicate cell proliferation assays (mean ± SEM) were analyzed by ANOVA and Tukey’s Test on the treatment means. Each flow cytometry study was repeated at least 3 times the data represent the mean ± SEM for three separate determinations analyzed by ANOVA and Tukey’s test on the treatment means.

### Abbreviations

The EGFRSP genes subject to luteolin regulation included epidermal growth factor receptor (EGFR), v-fos FBJ marine osteosarcoma viral oncogene homolog (c-FOS), Son of sevenless homolog 1 (SOS), mitogen-activated protein kinase 8 (JNK1), growth factor receptor bound protein 2 (GRB2), mitogen-activated protein kinase 4 (MKK4), Ras-GTP-ase activating protein SH3-Domain Binding Protein (RasGAP). The CCP genes included cyclin-dependent kinase inhibitor 1B (CDKN1B; p27), cyclin A2 (CCNA2), polo-like kinase I (PLK1), cell division cycle 25A (CDC25A), cyclin E2 (CCNE2), proliferating cell nuclear antigen (PCNA), cyclin D1 (CCND1) and cyclin-dependent kinase inhibitor 1A  (CDKN1A; p21). Other abbreviations include ethanol (EtOH), dimethylsulfoxide (DMSO) and estrogen receptor (ERα or ERβ).

## RESULTS

### Effects of BMHPC on EGFRSP and CCP Genes in PC-3 Cells

A number of genes in the EGFRSP (EGFR, c-Fos, SOS, GRB2, JNK1, MKK4, RasGAP) and CCP (CCNA2, CCNE2, CDC25A, CDKN1A, p21, p27, PLK1) are regulated by luteolin in PC-3 cells (35, 37). Two of these genes, c-FOS and p21, were maximally stimulated (400-2000% of control) by luteolin treatment. BMHPC effects on the EGFRSP genes and p21 were not evaluated in this earlier study. Since luteolin and BMHPC are type II site ligands known to antagonize normal and malignant prostate cell (PC-3, DU-145, LNCaP) growth and proliferation (22, 23), one would predict both compounds would regulate EGFRSP and CCP genes in PC-3 prostate cancer cells in a very similar fashion. Primary effects on c-FOS and p21 were expected and these possibilities were addressed.

**Figure 1:**
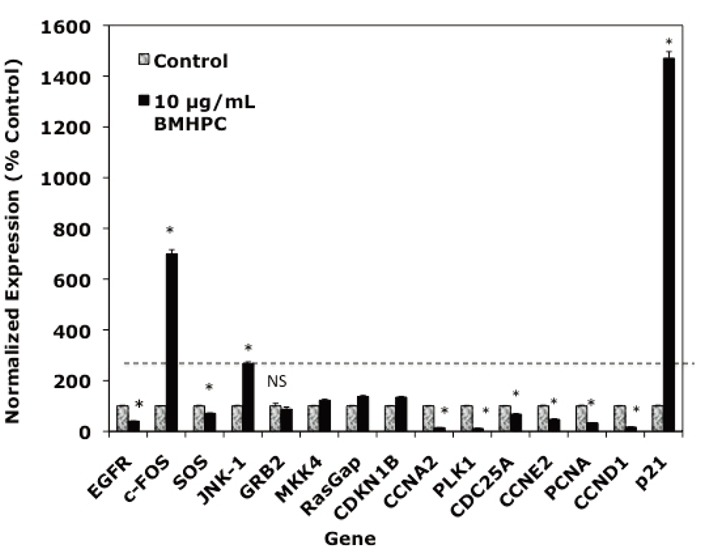
Effects of BMHPC on EGFSP and CCP Gene Expression in PC-3 Cells. Triplicate wells of exponentially growing PC-3 cells were treated at time 0 (24 hours following plating) with vehicle (controls) or 10 μg/mL BMHPC in vehicle for 24 hours. Cells were collected in RNA-Later and RNA prepared from the controls and BMHPC-treated cells was analyzed by QPCR for assessment of treatment effects on c-FOS or p21 gene expression normalized to 18S RNA. The QPCR values are the mean ± SEM for three independent RNA sets and are represented as normalized expression as a percent of the vehicle controls (control=100%).

Treatment of PC-3 cells with BMHPC for 24 hours (Figure 1) markedly stimulated c-FOS, JNK-1 and p21 gene RNA expression and suppressed the expression of EGFR, SOS, CCNA2, PLK1, CDC25A, CCNE2 and CCND1. Thus, the response to BMHPC at 24 hours (Figure 1) was essentially indistinguishable from their previously reported response profile to luteolin (37). The magnitude of the response of c-FOS and p21 to luteolin (35) or BMHPC (Figure 1) was substantial (400-1000% of control) relative to that of other genes in these pathways. This observation suggests that c-FOS and p21 RNA expression may be central to anti-proliferative activity of the two compounds. The remainder of the studies in this manuscript focused on c-FOS and p21 RNA expression to further define the roles of these two genes in the proliferative response of PC-3 cells to these type II site ligands.

### Reversibility of Response to Luteolin and BMHPC

**Figure 2:**
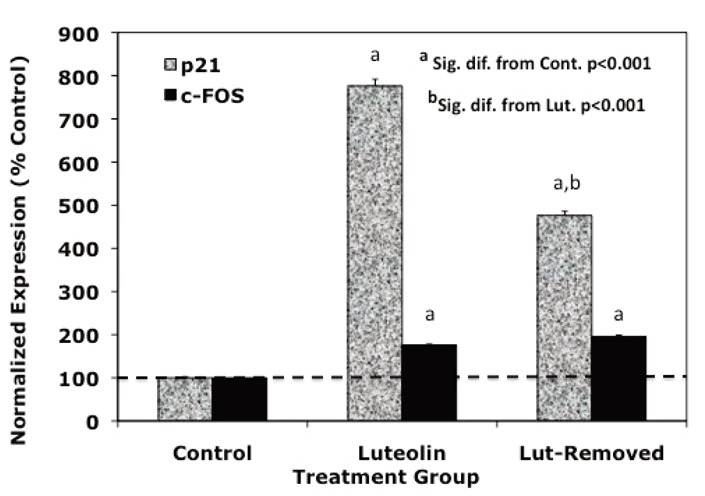
Effects of Luteolin Removal on c-FOS or p21 Gene Expression at 48 Hours in PC-3 Cells. Exponentially growing PC-3 cells were treated with vehicle (5 μL) or 10 μg/mL luteolin in vehicle at time 0 (24 hours following plating) and grown for an additional 48 hours in the presence of the bioflavonoid, or were subjected to a media change after 24 hours of luteolin treatment, and grown for an additional 24 hours in the absence of luteolin (Lut-Removed) prior to collection for RNA isolation. Treatment effects on c-FOS and p21 gene expression were determined by QPCR and normalized to 18S RNA. Data for each treatment group and time point were analyzed by ANOVA and Tukey’s test on the treatment means (Intstat, GraphPad Software) and are represented as the mean ± SEM for normalized expression relative to the vehicle controls (control=100%).

**Figure 3:**
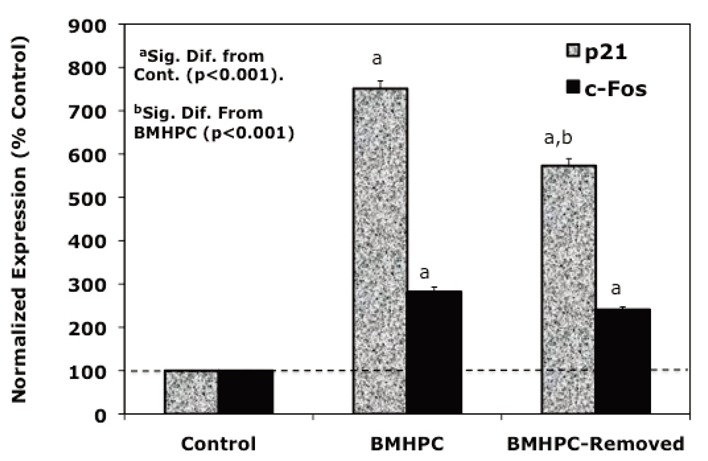
Effects of BMHPC-Removal on c-FOS or p21 Gene Expression at 48 Hours in PC-3 Cells. Exponentially growing PC-3 cells were treated with vehicle (5 μL) or 10 μg/mL BMHPC in vehicle at time 0 (24 hours following plating) and grown an additional 48 hours prior to RNA isolation and assessment of c-FOS or p21 gene expression by QPCR as described in Figure 1 and Materials and Methods. Cells in the BMHPC-removed group were subjected to a media change after 24 hours of BMHPC treatment and were grown an additional 24 hours in the absence of this ligand prior to collection for RNA isolation. BMHPC effects on c-FOS and p21 gene expression were analyzed by QPCR and normalized to 18S RNA (see Figure 1 and Materials and Methods for details). Data on triplicate wells for each treatment group and time point were analyzed by ANOVA and Tukey’s test on the treatment means (Instat, GraphPad Software) and represent the mean ± SEM of the normalized expression expressed as a percent of the control (control=100%).

Dose response and time studies (37) demonstrated that c-FOS and p21 RNA expression is maximally stimulated by type II ligands in PC-3 cells 24-48 hours following treatment. To further compare the response profiles of c-FOS and p21 RNA to the type II ligands, we assessed the effects of acute (drug removed after 24 hours of treatment) versus sustained (48 hours continuous treatment) treatment with luteolin (Figure 2) or BMHPC (Figure 3) on the expression of c-FOS or p21 RNA by QPCR. The magnitude of the response of c-FOS (200-800 % control; Figures 2 and 3) to either ligand was similar and sustained regardless of whether the cells were treated continuously or acutely with luteolin (Figure 2) or BMHPC (Figure 3). Although continuous luteolin or BMHC treatment markedly stimulated (700-800%) p21 RNA expression relative to vehicle controls, a significant decline in p21 RNA was observed 24 hours following the removal of the drugs from the media. Thus, p21 RNA expression was not sustained for as long as c-FOS RNA expression following the discontinuation of luteolin or BMHPC treatment. This may indicate that these two genes are independently controlled, a response contrary to that reported for c-FOS and p21 following the stimulation of GPR30 pathways in PC-3 cells (39). Alternatively, perhaps the RNA half-lives of these two genes is different which could account for this varied response.

### Flow Cytometry Studies

Luteolin and BMHPC effects on the proliferation of PC-3 cell proliferation were also assessed by flow cytometry utilizing PC-3 cells treated continuously with luteolin or BMHPC for 24, 48 or 72 hours, or at the indicated times following drug removal. The results were very similar for luteolin (Table 1) or BMHPC (Table 2). Both compounds caused irreversible G2/M arrest as reflected by a significant (*p*<0.01 to *p*<0.001) increase in the number of cells in G2/M at 24, 48 or 72 hours following treatment. As expected, the G2/M block was reflected by significant decreases (*p*<0.001) in the numbers of PC-3 cells in G0/G1, and significant (*p*<0.01 to *p*<0.001) increases in dead cells persisting for 24-hours following the removal of luteolin or BMHPC from the culture media. Thus, the irreversible “cell cycle responses” to these compounds were very similar, suggesting a common mechanism of action.

**Table 1:** Luteolin Effects on PC-3 Cell Cycle

Treament	G0/G1	S	G2/M	Sub G0

24 Hr Control	61.6 ± 0.4	11.6 ± 0.6	22.5 ± 0.6	1.5 ± 0.1
24 Hr Luteolin	49.3 ± 1.4^c^	12.7 ± 1.3	31.2 ± 0.9^b^	3.4 ± 0.2
24 Hr Control Removed for 24 Hr	65.9 ± 0.8	8.5 ± 0.3	22.1 ± 0.4	1.4 ± 0.03
24 Hr Luteolin Removed for 24 Hr	39.3 ± 0.7^c^	10.6 ± 0.3	29.0 ± 0.9^a^	8.8 ± 1.2^c^
24 Hr Control Removed for 48 Hr	83.7 ± 0.7	3.5 ± 0.4	8.8 ± 0.7	2.7 ± 0.4
24 Hr Luteolin Removed for 48 Hr	47.5 ± 0.5^c^	8.5 ± 0.5^a^	24.3 ± 0.4^c^	9.4 ± 1.1^b^
24 Hr Control Removed for 72 Hr	86 ± 1.4	2.8 ± 0.5	8.0 ± 0.7	1.6 ± 0.03
24 Hr Luteolin Removed for 72 Hr	54.4 ± 1.0^c^	7.6 ± 0.9^a^	24.7 ± 0.8^c^	6.4 ± 0.2

a Significantly Different from relevant control (*p*<0.05);

b Significantly Different from relevant control (*p*<0.01);

c Significantly Different from relevant control (*p*<0.001).

**Table 2:** BMHPC Effects on PC-3 Cell Cycle

Treament	G0/G1	S	G2/M	Sub G0

24 Hr Control	61.6 ± 0.4	11.6 ± 0.6	22.5 ± 0.6	1.5 ± 0.1
24 Hr BMPHC	43.1 ± 0.5^c^	11 ± 1.8	39.3 ± 3.0^c^	2.6 ± 0.1
24 Hr Control Removed for 24 Hr	65.9 ± 0.8	8.5 ± 0.3	22.1 ± 0.4	1.4 ± 0.03
24 Hr BMPHC Removed for 24 Hr	32.6 ± 2.5^c^	10.9 ± 0.9	36.4 ± 1.1^c^	8.4 ± 2.9^b^
24 Hr Control Removed for 48 Hr	83.7 ± 0.7	3.5 ± 0.4	8.8 ± 0.7	2.7 ± 0.4
24 Hr BMPHC Removed for 48 Hr	27.8 ± 0.7^c^	7.1 ± 1.1	40.5 ± 2.0^c^	12.7 ± 0.3^c^
24 Hr Control Removed for 72 Hr	86 ± 1.4	2.8 ± 0.5	8.0 ± 0.7	1.6 ± 0.03
24 Hr BMPHC Removed for 72 Hr	32.4 ± 0.3^c^	6.3 ± 0.6^a^	30.1 ± 0.5^c^	24.3 ± 0.6^c^

a Significantly Different from relevant control (*p*<0.05);

b Significantly Different from relevant control (*p*<0.01);

c Significantly Different from relevant control (*p*<0.001).

### Effects of c-FOS and p21 siRNA Knockdown on Luteolin and BMHPC Inhibition of PC-3 Cell Proliferation

**Figure 4:**
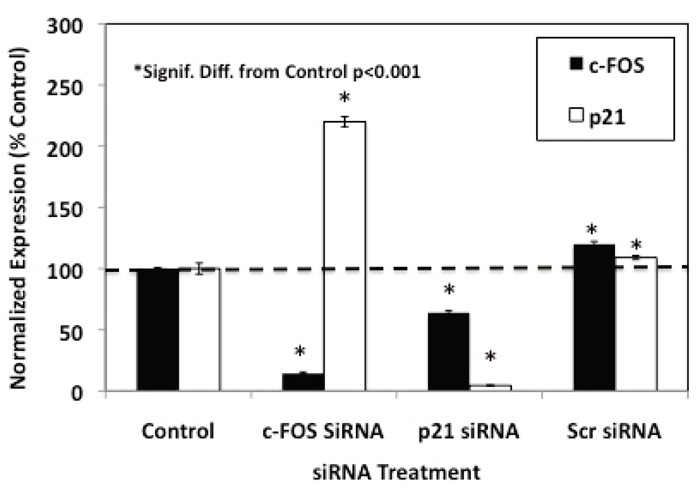
siRNA Knockdown of c-FOS and p21 Genes in PC-3 Cells. Triplicate wells of exponentially growing PC-3 cells for each experimental group were treated with Lipofectamine 2000 (8 μL/flask; controls) or 30 nM c-FOS siRNA for, p21 siRNA or Scr siRNA (non-Target scrambled siRNA) from Dharmacon for 48 hours. At this time, the cells were collected in RNA-Later (Qiagen) and RNA was prepared for QPCR analysis of c-Fos and p21 RNA expression as described in Materials and Methods. Results from triplicate pools of RNA for each treatment group were normalized to 18S RNA and data expressed as per cent of the Lipofectamine control (100%). Data were analyzed statistically by ANOVA and Tukey’s test on the treatment means and expressed as the mean ± the SEM.

The flow cytometry studies suggested that luteolin or BMHPC inhibit PC-3 cell proliferation through a common mechanism involving the regulation of c-FOS and/or p21 gene expression and G2/M arrest. This finding is consistent with the association of these two genes in the control of PC-3 cell proliferation (39). To further define the roles of c-FOS and p21 in the overall mechanism of action of luteolin or BMHPC, siRNA knockdown studies were done. Preliminary time and dose studies (not shown) indicated that maximum target gene knockdown (RNA and protein) was obtained following 48 hours of pre-treatment with 30 nM concentrations of the c-FOS or p21 siRNA. These conditions were used in the studies shown in Figure 4. Treating PC-3 cells for 48 hours with 30 nM c-FOS siRNA or 30 nM p21 siRNA resulted in significant knockdown (»90%) of their respective target genes. Treatment with the 30 nM non-target (scrambled) siRNA failed to inhibit the expression of either c-FOS or p21. It is important to note that the observed c-FOS RNA knockdown with c-FOS siRNA was associated with a significant (*p*<0.001) enhancement of p21 RNA expression, and likewise, the p21 knockdown with p21 siRNA correlated with increased (*p*<0.001) c-FOS RNA expression confirming a close relationship between the two genes in PC-3 cells (37, 39).

**Figure 5:**
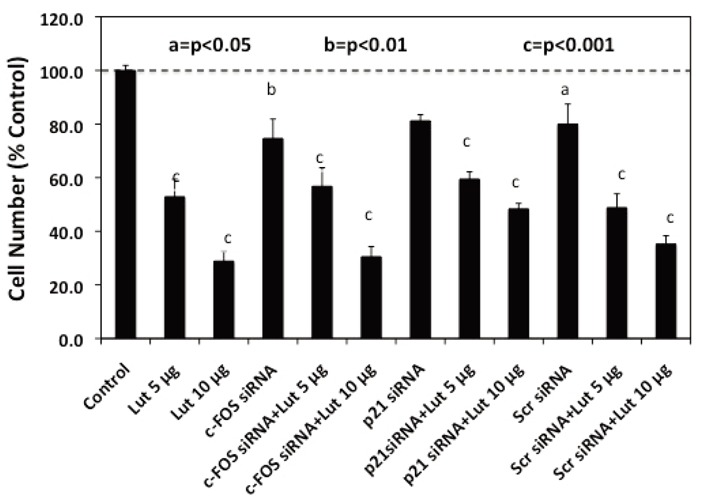
Effects of c-FOS and p21 Knockdown on Luteolin Inhibition of PC-3 Cell Proliferation. Triplicate wells of exponentially growing PC-3 cells for each treatment group were treated with Lipofectamine 2000 (Control), or 30 nM concentrations of c-FOS siRNA, p21 siRNA or Scr siRNA on Day 0 (24 hours following plating) and grown for an additional 48 hours in the presence of 4-8 μL Lipofectamine 2000 (Control) or the various siRNA’s added in Lipofectamine 2000 as described in Materials and Methods. At this time (48 hrs following siRNA treatment), the cells were treated with 5 μL vehicle (control) or 5 or 10 μg/mL luteolin (Lut) added to the medium in 5 μL of ethanol and grown for an additional 96 hours prior to cell collection and counting by crystal blue dye uptake. Data were analyzed statistically by ANOVA and Tukey’s test on the treatment means and results are expressed as the mean ± SEM relative to the vehicle control (100%).

**Figure 6:**
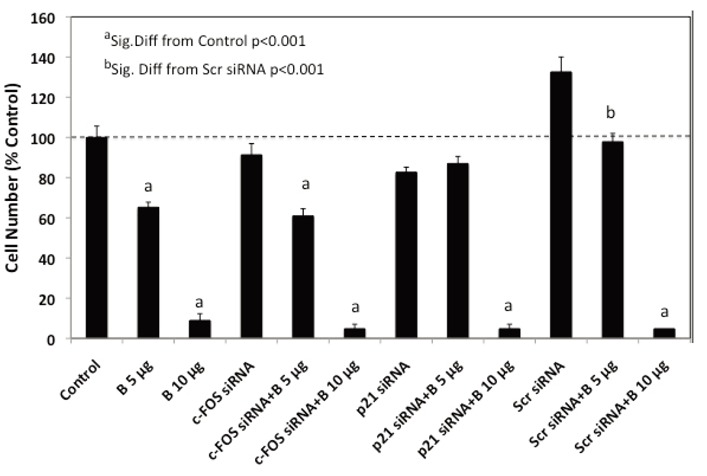
Effects of c-FOS and p21 Knockdown on BMHPC Inhibition of PC-3 Cell Proliferation. Triplicate wells of exponentially growing PC-3 cells for each treatment group were treated with Lipofectamine 2000 (Control), or 30 nM concentrations of c-FOS siRNA, p21 siRNA or Scr siRNA on Day 0 as described in Figure 7. Forty-eight hours following siRNA treatment, the cells were treated with 5 μL vehicle (Control) or 5 or 10 μg/mL BMHPC (B) added to the medium in 5 μL of vehicle and grown for an additional 96 hours prior to cell collection and counting by crystal blue dye uptake. Data were analyzed statistically by ANOVA and Tukey’s test on the treatment means. Results are expressed as the mean ± SEM relative to the vehicle control (100%).

To quantify changes in RNA expression as they are related to downstream luteolin or BMHPC modulation of cell proliferation, we assessed the effects of c-FOS and p21 siRNA treatment on luteolin (Figure 5) or BMHPC (Figure 6) inhibition of PC-3 cells. The cells were pre-treated for 48 hours with c-FOS siRNA or p21 siRNA to achieve the 90-95% knockdown of the two target genes shown in Figure 4. Companion studies with non-target (scrambled) siRNA served as additional controls. Following vehicle (controls) or siRNA pre-treatment, the cells were then treated for an additional 96 hours with luteolin (Figure 5) or BMHPC (Figure 6). Cell numbers were determined at this time (144 hours of the study). Pre-treatment with either c-FOS or p21 siRNA’s for 48 hours (reduces c-FOS or p21 RNA by »90%) did not block luteolin or BMHPC inhibition of PC-3 cell proliferation. The dose response profiles to luteolin or BMHPC were similar to controls in siRNA treated cells and the scrambled siRNA failed to substantially alter the anti-proliferative response to either luteolin or BMHPC. Thus, 90-95% knockdown of c-FOS or p21 RNA expression did not substantially block cell proliferation or alter the anti-proliferative response to luteolin or BMHPC.

### Combination siRNA Knockdown of c-FOS and p21 in PC-3 cells

**Figure 7:**
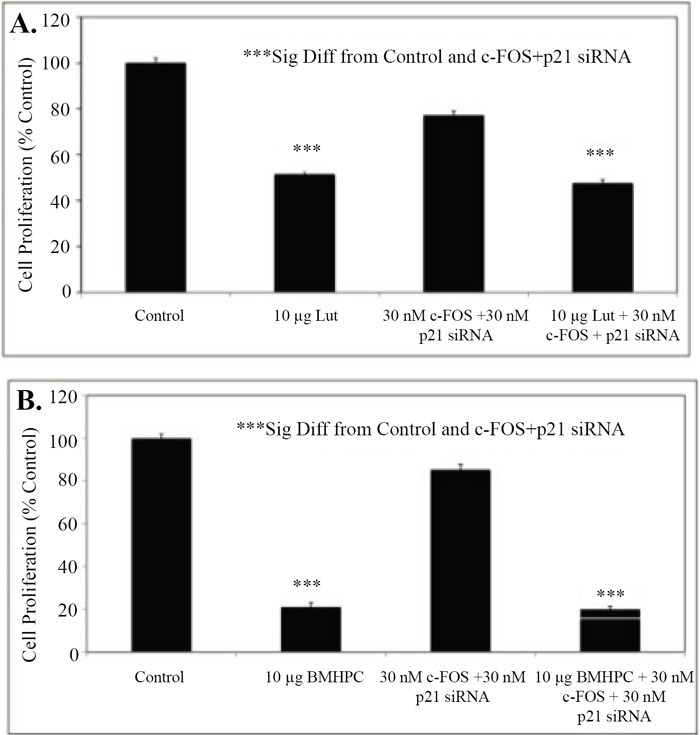
Effects of Combined c-FOS and p21 Knockdown on Luteolin Inhibition of PC-3 Cell Proliferation. Triplicate wells of exponentially growing PC-3 cells for each treatment group were treated with Lipofectamine 2000 (Control), or 30 nM c-FOS + 30 nM p21 siRNA on Day 0 as described in Figures 5 and 6. Forty-eight hours following siRNA addition, the cells were treated with 10 μg/mL luteolin (Lut; Figure 7A) or BMHPC (Figure 7B) added to the medium in vehicle and grown for an additional 72 hours (BMHPC) or 96 hours (luteolin) prior to harvesting and counting in Trypan Blue. Data were analyzed statistically by ANOVA and Tukey’s test on the treatment means. Results are expressed as the mean ± SEM relative to the vehicle control (100%).

The data in Figures 4-6 show that individual treatment with siRNA’s for c-FOS or p21 RNA did not block luteolin or BMHPC inhibition of PC-3 cell proliferation. Previous studies with luteolin (37), and those presented here for BMHPC (Figure 1), indicate that the response of PC-3 cells to either ligand is characterized by a major stimulation (400-2000% of control) of c-FOS and p21. The siRNA studies noted above suggest significant interplay between c-FOS and p21 may alter the RNA expression response to luteolin and BMHPC. If the inhibitory response to luteolin or BMHPC is mediated only through the modulation of RNA expression by these two genes, the double knockout should completely ablate the response to the type II site ligands. Therefore, PC-3 cells were treated with vehicle (controls) or 30 nM concentrations of c-FOS and p21 siRNA (Combo) for 48 hours. At this time, the cells were further treated with vehicle or 10 μg/mL luteolin or 10 μg/mL BMHPC for and additional 72 hours (BMHPC) or 96 hours (luteolin) according to the protocol in Figures 5 and 6. Thus, the total experimental period for BMHPC and luteolin was 120 or 144 hours, respectively. The treatment period for BMHPC was decreased from 96 hours (Figure 6) to 72 hours (Figure 7B) because the longer BMHPC treatment period (Figure 6) nearly completely inhibited cell proliferation. More cells were desired for counting purposes so the 72 hour treatment period was used (Figure 7B) for assessment of BMHPC effects on cell proliferation in the combination siRNA studies. The 96-hour treatment period (Figure 5) was suitable for the luteolin study (Figure 7A). BMHPC is more non-polar than luteolin and more rapidly adsorbed by the cells. In either case, luteolin (Figure 7A) or BMHPC (Figure 7B) significantly (*p*<0.001) inhibited the proliferation of PC-3 cells. We attempted to perform western blots for the proteins at these longer times, but were unable to collect significant numbers of cells for these analyses.

Treatment of PC-3 cells with a combination of 30 nM concentrations of the siRNA’s for c-FOS + p21 only slightly suppressed cell proliferation (10-20%) relative to the vehicle controls (100%). Nevertheless, the combined siRNA’s failed to block luteolin (Figure 7A) or BMHPC (Figure 7B) inhibition (*p*<0.001) of PC-3 cell proliferation. The inhibitory response to luteolin (Figure 7A) or BMHPC (Figure 7B) was nearly identical in absence or presence of siRNA’s to c-FOS and p21. Thus, although c-FOS and p21 RNA expression is modulated by luteolin or BMHPC in PC-3 cells, it is possible that other genes in the EGFSP and CCP (Figure 1) independently regulated by luteolin or BMHPC are involved in the anti-proliferative response to these type II site ligands under conditions where c-FOS and p21 expression are blocked.

**Figure 8:**
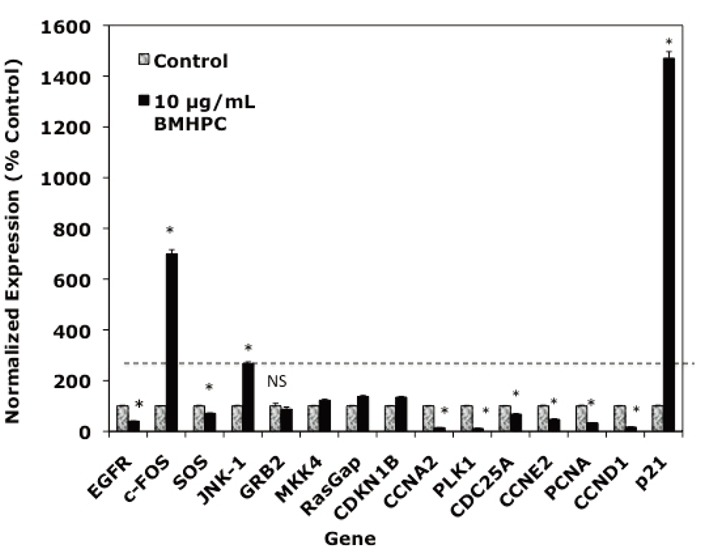
Effects of Combined c-FOS and p21 siRNA (Combo) Knockdown on c-FOS and p21 Gene Expression in PC-3 Cells. Triplicate wells of exponentially growing PC-3 cells for each experimental group were treated with Lipofectamine 2000 (8 μL/ flask; controls) or 30 nM c-FOS siRNA or p21 siRNA for 48 hours. At this time, the cells were treated for an additional 24 hours with vehicle (control) or 10μg/mL of luteolin (Panel A) or BMHPC (Panel B) to assess the Combo siRNA effects on luteolin or BMHPC induction of c-FOS or p21. RNA was prepared for QPCR analysis of c-Fos and p21 RNA expression as described in Materials and Methods. Results from triplicate pools of RNA for each treatment group were normalized to 18S RNA and data expressed as per cent of control (100%). Data were analyzed statistically by ANOVA and Tukey’s test on the treatment means and expressed as the mean ± the SEM. Equivalent results were obtained when RNA was prepared from cells treated for longer times (72-96 hours) with luteolin (Figure 7A) or BMHPC (Figure 7B) plus or minus the Combo siRNA’s (data not shown). ^a^Significanly Different from Control (*p*<0.001); ^b^Significantly Different from BMHPC (*p*<0.001).

QPCR analysis on RNA from cells treated with the siRNA Combo and/or luteolin or BMPHC (Figure 8) confirmed the siRNA inhibition (Figure 4) and cell proliferation data (Figure 7). Consistent with the data in Figures 2 and 3, both luteolin (Figure 8A) and BMHPC (Figure 8B) significantly (*p*<0.001) stimulated c-FOS expression relative to the control. BMHPC (Figure 8B) also stimulated p21 RNA expression under these conditions in a manner similar to that shown in Figures 1 and 3. The inability of luteolin to stimulate p21 RNA expression under these conditions (Figure 8A), as opposed to those in Figure 2, was attributed to Lipofectamine 2000 suppression of this response. Lipofectamine and other transfection reagents are known to interfere with cell response. In the absence of Lipofectamine 2000 the full stimulatory response of p21 to luteolin (»800% increase above control; Figure 2) is observed. Lipofectamine 2000 suppression of luteolin stimulation of p21 RNA expression (Figure 8A) is specific to the interaction of luteolin, Lipofectamine 2000 and p21 because luteolin stimulated c-FOS expression in the presence of Lipofectamine 2000 (Figure 8A). Similarly, BMHPC stimulated both c-FOS and p21 RNA expression in the presence of the Lifectamine 2000 (Figure 8B). The lack of p21 RNA response to luteolin in this experiment is inconsequential in that the purpose of the Combo siRNA study with siRNA’s to c-FOS and p21 was to evaluate the effects of luteolin and BMHPC on cell proliferation under conditions where c-FOS and p21 RNA expression are reduced. This was accomplished. The data in Figure 8 show that treatment with the Combo siRNA’s significantly (*p*<0.001) reduced both c-FOS and p21 RNA expression (Figures 8A and 8B) to very low levels in the absence (Combo alone; Figures 8A and 8B) or presence of luteolin (Lut + Combo; Figure 8A) or BMHPC (BMHPC + Combo; Figure 8B). In fact, the stimulatory response of either c-FOS or p21 to luteolin (Figure 8A) or BMHPC (Figure 8B) was nearly completely blocked (*p*<0.001) by the Combo siRNA’s (Lut + Combo Figure 8A or BMHPC + Combo Figure 8B) relative to the cells treated with luteolin (Lut; Figure 8A) or BMHPC (BMHPC; Figure 8B) alone. Thus, the Combo siRNA’s largely ablated luteolin or BMPHC stimulation of c-FOS or p21 siRNA expression (Figure 8) even though such treatment failed to block luteolin or BMHPC inhibition of PC-3 cell proliferation (Figure 7). These data further confirm the notion that the effects of luteolin or BMHPC on cell proliferation are not totally mediated through of c-FOS and p21. Other factors (genes) are likely involved in the anti-proliferative response to these agents.

## DISCUSSION

A goal of our laboratory is to define the role of nuclear type II sites and their ligands in the regulation of normal and abnormal cell growth and proliferation. Interaction of MeHPLA with the nuclear type II site is a component of an important growth regulatory pathway in mammalian cells. Occupancy of type II sites by MeHPLA inhibits cell proliferation (23). The enzymatic hydrolysis of MeHPLA to HPLA represents a normal response in non-malignant tissues, and is likely responsible for the loss of regulatory control in malignant cells. HPLA does not bind to nuclear type II sites with sufficient affinity to inhibit normal or malignant cell proliferation *in vitro* or *in vivo* (15, 16, 21, 24, 25, 41). Consequently, esterase-stable ligands (luteolin, BMHPC) that mimic MeHPLA as cell growth regulating agents inhibit normal and malignant cell proliferation *in vivo* and *in vitro* in a variety of experimental systems(4, 14, 20, 22, 23, 26, 28, 29, 38, 42-48).

The identification of the nuclear type II site as histone H4 provided insight into the role of this protein and its ligands in mammalian cell function (31-33, 49). This finding suggests that MeHPLA and related ligands control specific gene transcription via an epigenetic mechanism involving histone H4 modifications (acetylation/deacetylation, methylation, phosphorylation, ubiquination, etc.) that up- or down-regulate gene transcription (50-52). This notion is supported by cRNA microarray and QPCR and studies identifying genes in the estrogen-signaling pathway, RNA transcription pathways, EGFRSP and CCP in breast and prostate cancer cells that are regulated by luteolin and BMHCP (53, 54). Chromatin immunoprecipitation (ChiP) studies suggest this occurs via the acetylation of histone H4 at the promoter level (34, 35, 37). That histone H4 (type II sites) are ubiquitous suggest this pathway could be targeted for drug development to treat proliferative diseases of all mammalian cells.

The studies in this manuscript address a number of specific questions relating to the regulation of specific genes in the EGFRSP and CCP by type II site ligands. Luteolin and BMHPC bind to type II sites with high affinity in nuclear preparations of normal prostate (22) and in androgen-dependent (LNcaP) and androgen-independent (PC-3, DU-145) and human prostate cancer cells (23). Both compounds inhibit the proliferation of these cells *in vitro* and *in vivo* and both luteolin (35, 37) and BMPHC (Figure 1) regulate the expression of genes in the EGFRSP (EGFR, SOS, GRB2, JNK1, MKK4, RasGAP) and CCP (CCNA2, CCNE2, CDC25A, CDKN1A, CDKN1B, p27, PLK1) in addition to c-FOS and p21. These observations are consistent with the abilities of these type II site ligands to inhibit PC-3 cell proliferation.

The effects of both ligands on c-FOS and p21 RNA were of prime interest because the expression of these two genes is most markedly stimulated by luteolin (37) or BMHPC (Figure 1) relative to the other genes in the ESFRSP or CCP. Thus, the up-regulation of c-FOS and/or p21 seemed central to effects of the two ligands on PC-3 cell proliferation. Even though luteolin and BMHPC are structurally very different compounds (22, 23), they mirror each other in terms of their binding affinities for nuclear type II sites (22, 23), modulation of EGFRSP and CCP genes (Figure 1), reversible effects on c-FOS and p21 gene expression (Figures 2 and 3) and their effects on PC-3 cell cycle dynamics including the irreversible G2/M arrest (Tables 1 and 2). These findings are consistent with the hypothesis that luteolin and BMHPC regulate gene expression through a common mechanism likely mediated by binding to histone H4 (31, 32, 49). Direct involvement of histone H4 in this process is not possible to prove since the knockout of this gene is lethal.

We also assessed the effects of siRNA’s to c-FOS or p21 on luteolin or BMHPC stimulation of these two genes (RNA expression) and the inhibition of PC-3 cell proliferation. Pretreatment of PC-3 cells with siRNA’s to c-FOS and p21 for 48 hours resulted in 90-95% knockdown of their respective RNA’s (Figure 4). However, neither siRNA blocked luteolin (Figure 5) or BMHPC (Figure 6) inhibition of PC-3 cell proliferation. The cell inhibitory dose response to the either luteolin or BMHPC was essentially identical in controls (vehicle treated cells) or in cells pre-treated with scrambled siRNA, c-FOS siRNA or p21 siRNA (Figures 5 and 6). The double knockdown studies with both siRNA’s also failed to alter the ability of either luteolin or BMHPC to inhibit cell proliferation (Figures 7A and 7B) even though the combination siRNA’s nearly completely blocked of cFOS or p21 RNA expression in the absence or presence of luteolin (Figure 8A) or BMHPC (Figure 8). These findings support the concept that luteolin and BMHPC inhibition of PC-3 cell proliferation can occur independent of their effects on c-FOS or p21 RNA expression and therefore, c-FOS and p21 may only play supportive roles in the overall response. The data do not rule out the possibility that c-FOS or p21 are components of the overall inhibitory response to luteolin or BMHPC, but they certainly suggest that the other genes in the EGFRSP or CCP modulated by luteolin and BMHPC (see Figure 1), or other unknown mechanisms, are involved in this anti-proliferative response. It is clear that luteolin modulates the acetylation of H4 at the PLK1 promoter in PC-3 cells (35) and luteolin and BMHPC inhibition of PLK1 expression is associated with the down-regulation of CDC25B, CDC25C, and CDC45L RNA expression in PC-3 cells (37). The latter two genes are M-phase inducer phosphatases that trigger entry into mitosis and CDC45L is involved in the initiation of DNA replication (55-57). This notion is consistent with luteolin and BMHPC induction of G2/M arrest in PC-3 cells (Tables 1 and 2). Therefore, The G2/M arrest caused by luteolin and BMHPC treatment may be mediated at the level of the PLK-1 promoter. This is currently under investigation.

On the basis of the above observations, epigenetic regulation of some or all of the luteolin or BMHPC regulated genes shown in Figure 1 for BMHPC and luteolin (37) is suspect. What determines which histone H4 molecules (type II sites) on target gene promoters are available for binding these inhibitory ligands remains a major unanswered question. Of the 38,500 genes monitored in our microarray studies with the Human Genome U133 Plus 2.0 oligonucleotide arrays (35), luteolin regulated the expression of about 3000 genes. Thus, 7% of the genes in PC-3 cells are responsive to type II site ligands. If our original hypothesis is correct, perhaps only certain gene promoters in proliferating cells which have unoccupied type II sites are capable of binding these inhibitory ligands. Type II sites are occupied by MeHPLA in non-proliferating tissues and cell proliferation is held to a basal level (23). Mitogenic stimulation causes a dissociation of MeHPLA from nuclear type II sites, MeHPLA hydrolysis to HPLA, and a net increase of unoccupied type II sites available for ligand binding (13-16). If this model is correct, the promoters of genes regulated by luteolin (35, 37) or BMHPC (Figure 1) likely contain unoccupied type II sites. More detailed studies are required to identify molecular factors specifying which “type II site/promoters” are capable of binding ligands, and what biochemical- and/or molecular-modifications occur on these promoters following ligand binding to affect gene transcription. We are running ChiP (34, 35, 37) and re-Chip (58) assays to define luteolin and BMHPC effects on specific histone H4 tail modifications (acetylation/methylation/phosphorylation/ubiquination) on the promoters of the EGFRSP and CCP genes identified in these studies (Figure 1) and the recruitment of transcription factors to these promoters (59-61).
